# Patient with a retained bullet near the carotid bifurcation: simultaneous carotid endarterectomy and beating-heart LIMA–LAD bypass in a 70-year-old man

**DOI:** 10.1097/RC9.0000000000000495

**Published:** 2026-04-27

**Authors:** Ferid Gojayev, Anıl Güzeloğlu

**Affiliations:** Department of Cardiovascular Surgery, Koşuyolu High Specialization Training and Research Hospital, Istanbul, Türkiye

**Keywords:** carotid bifurcation, carotid endarterectomy, LIMA–LAD, off-pump coronary artery bypass, retained bullet, simultaneous revascularization

## Abstract

**Introduction and importance:**

Significant carotid artery stenosis (CAS) in patients requiring coronary artery bypass grafting (CABG) confers a combined risk of perioperative stroke and myocardial ischemia. Management must therefore be individualized, particularly in the presence of uncommon anatomical challenges such as retained ballistic fragments adjacent to major cervical vessels. We report a rare case of synchronous carotid endarterectomy (CEA) with bullet extraction followed by off-pump CABG.

**Case presentation:**

A 70-year-old man presented with exertional chest pain and dyspnea. Coronary angiography demonstrated an 85% proximal left anterior descending (LAD) artery stenosis. Preoperative carotid duplex ultrasonography and computed tomography angiography revealed approximately 90% stenosis of the right internal carotid artery, associated with a metallic foreign body at the carotid bifurcation consistent with a retained bullet from a gunshot injury sustained 30 years earlier. After multidisciplinary evaluation, a simultaneous surgical strategy was adopted. The patient underwent right CAE with careful extraction of the ballistic fragment, immediately followed by off-pump (beating-heart) left internal mammary artery to LAD bypass. The postoperative course was uneventful, with early extubation and no neurological deficits. Follow-up duplex ultrasonography confirmed a widely patent carotid reconstruction.

**Clinical discussion:**

Patients with coexisting high-grade CAS and critical coronary artery disease require careful balancing of cerebrovascular and cardiac risks. In selected cases, synchronous CEA and CABG may be an appropriate strategy. An off-pump coronary approach may further reduce embolic risk by avoiding aortic manipulation, particularly in patients with significant carotid pathology. Retained bullets near the carotid bifurcation are exceptionally rare and substantially increase operative complexity due to fibrosis, altered tissue planes, and proximity to neurovascular structures.

**Conclusion:**

This case illustrates that simultaneous CEA with ballistic fragment extraction followed by off-pump LIMA–LAD bypass can be considered in carefully selected patients. The presence of a long-standing retained bullet at the carotid bifurcation highlights the importance of meticulous preoperative planning, detailed anatomical assessment, and multidisciplinary decision-making.

## Introduction

Concomitant severe carotid artery stenosis (CAS) in patients undergoing coronary artery bypass grafting (CABG) is associated with an increased perioperative stroke risk. The 2023 ESVS guidelines advocate individualized decision-making based on patient symptoms, stenosis severity, and urgency of cardiac surgery. For selected high-risk patients, synchronous carotid endarterectomy (CEA) and CABG may be considered. In coronary revascularization, off-pump CABG techniques have been shown to reduce early postoperative stroke, particularly in patients with cerebrovascular vulnerability. To our knowledge, reports describing synchronous CEA with removal of a retained ballistic fragment followed by off-pump CABG are extremely limited. The present case highlights the feasibility and technical considerations of this combined approach.

HIGHLIGHTS

- Retained bullet located directly beneath the carotid bifurcation complicated surgical planning.
- Simultaneous carotid endarterectomy (CEA) and off-pump LIMA–LAD bypass was safely performed in a single session.
- Off-pump strategy minimized embolic risk in a patient with severe unilateral carotid stenosis.
- Bullet extraction and CEA were executed without neurological deficit or perioperative morbidity.
- This case demonstrates a rare, complex vascular–cardiac hybrid approach with excellent outcomes.

## Case presentation

This work has been reported in accordance with the SCARE (Surgical CAse REport) 2025 guidelines^[^1^]^. A 70-year-old man was admitted with exertional chest pain and shortness of breath. His cardiovascular risk factors included advanced age and male sex; there was no prior history of stroke or transient ischemic attack. Electrocardiography showed ischemic changes, while high-sensitivity troponin levels were within normal limits. Coronary angiography revealed an 85% proximal stenosis of the left anterior descending (LAD) artery. The chronological sequence of clinical events is summarized in Table 1.

**Table 1:** Timeline of clinical course.

Timepoint	Clinical event
Presentation	Exertional chest pain and dyspnea
Initial evaluation	ECG with ischemic changes; normal high-sensitivity troponin
Coronary imaging	Coronary angiography showing 85% proximal LAD stenosis
Cerebrovascular imaging	Carotid duplex ultrasound and CTA revealing ~90% right ICA stenosis with metallic foreign body at the carotid bifurcation
Clinical history	Remote gunshot wound to the neck 30 years earlier
Multidisciplinary decision	Heart team decision for simultaneous carotid endarterectomy with bullet extraction and off-pump LIMA–LAD bypass
Surgical intervention	Right CEA with bullet extraction followed by off-pump LIMA–LAD bypass
Immediate postoperative course	Extubation at 4 hours; no neurological deficit
Discharge	Hemodynamically stable; neurologically intact
30-day follow-up	Asymptomatic; normal neurological examination; patent carotid reconstruction on duplex ultrasound

CEA, carotid endarterectomy; CTA, computed tomography angiography; ECG, electrocardiogram; ICA, internal carotid artery; LAD, left anterior descending artery; LIMA–LAD, left internal mammary artery to left anterior descending artery bypass.

**Figure 1.**
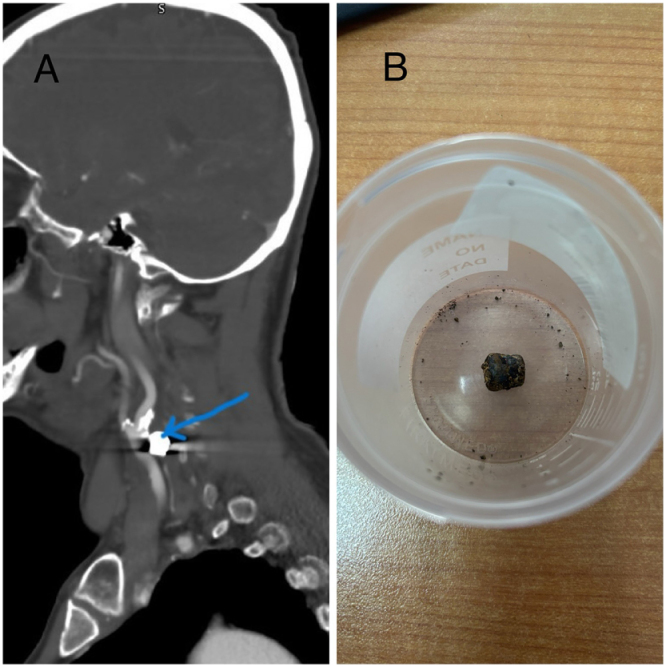
(A) Preoperative CTA showing critical right ICA stenosis (90%) with a metallic artifact near the carotid bifurcation (patient image). (B) İntraoperative photograph of the extracted bullet fragment after removal from beneath the ICA–ECA bifurcation (patient image).

As part of the preoperative evaluation, carotid duplex ultrasound and computed tomography angiography (CTA) demonstrated critical (approximately 90%) stenosis of the right internal carotid artery (ICA) accompanied by a metallic artifact near the carotid bifurcation (Fig. 1A). Upon further questioning, the patient recalled sustaining a gunshot wound to the neck 30 years earlier, suggesting a retained bullet fragment. Imaging localized the metallic object just below the ICA–external carotid artery (ECA) bifurcation and superficial to the vagus nerve (Fig. 1B).

In light of these findings – critical unilateral CAS without previous neurological symptoms but in the context of planned coronary surgery, the presence of a metallic foreign body adjacent to the bifurcation, and isolated severe LAD disease amenable to left internal mammary artery (LIMA) grafting – the multidisciplinary heart team decided to perform a simultaneous right CEA with bullet extraction, immediately followed by off-pump (beating-heart) LIMA–LAD bypass^[^1,2^]^.

## Operative details

Under general anesthesia, continuous cerebral monitoring with near-infrared spectroscopy (NIRS) was employed. The procedure was planned as a single-session hybrid operation with careful coordination between the cervical and cardiac phases.

The operation was initiated with a median sternotomy, and the LIMA was harvested in a pedicled fashion. Importantly, the distal end of the LIMA was not divided at this stage. The LIMA was intentionally harvested before systemic heparinization in order to allow dissection in a relatively bloodless field and to facilitate meticulous control of chest wall bleeding under optimal conditions. After careful hemostasis of minor bleeding sites along the internal chest wall, attention was redirected to the cervical field.

**Figure 2.**
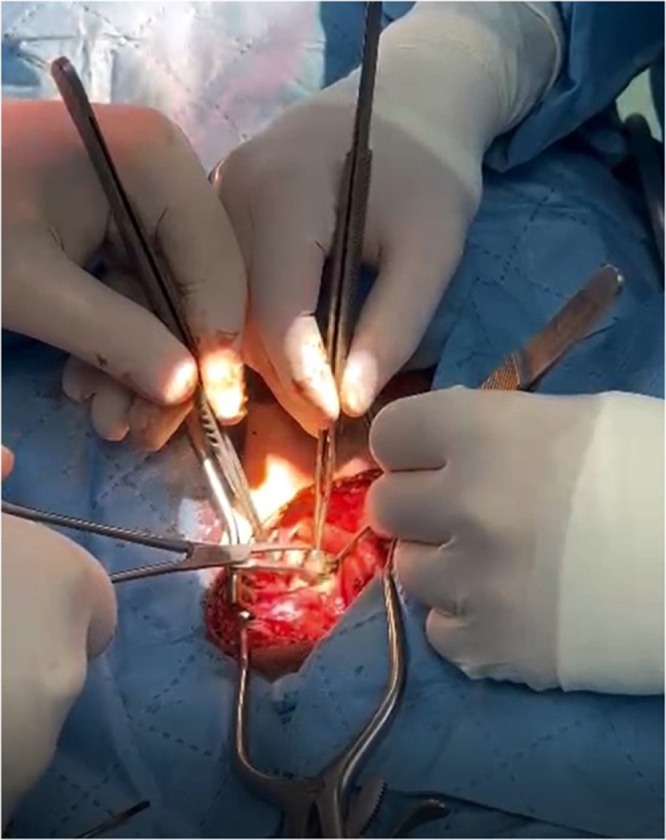
Intraoperative field showing the carotid bifurcation after bullet extraction and exposure of the ICA and ECA (patient image).

Following repositioning, systemic heparinization was administered, and the carotid phase was commenced. A longitudinal incision was made along the anterior border of the sternocleidomastoid muscle to expose the carotid sheath. Dense fibrotic tissue and altered dissection planes were encountered around the carotid bifurcation, consistent with chronic inflammatory changes related to the long-standing retained ballistic fragment. The bullet fragment was identified beneath the carotid bifurcation and superficial to the vagus nerve, carefully dissected, and removed *en bloc* (Fig. 2). The surgical procedure and intraoperative findings are demonstrated in Supplemental Digital Content Video 1, available at: http://links.lww.com/IJSCR/A39.

**Figure 3.**
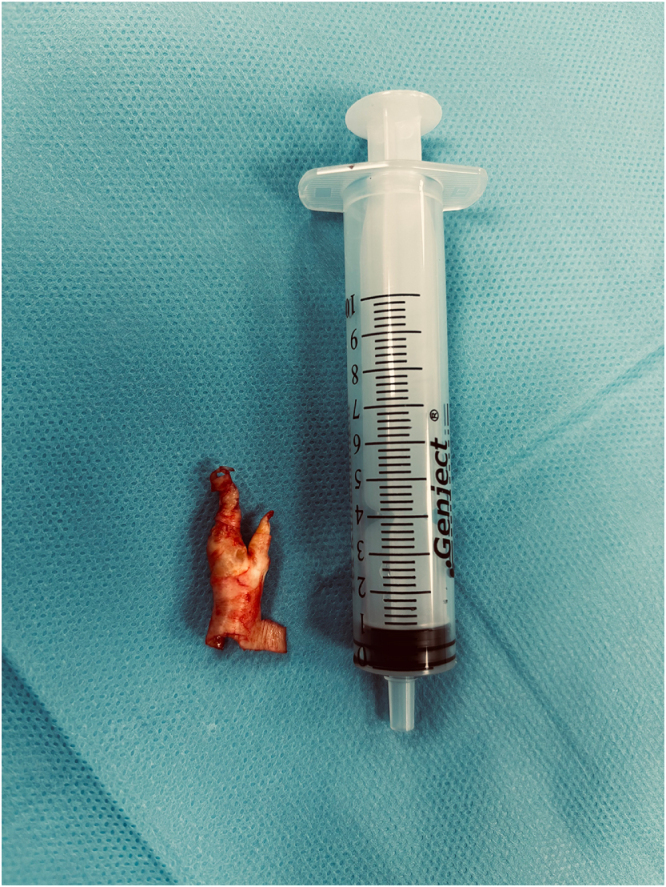
Carotid endarterectomy and patch angioplasty site prior to proceeding with coronary bypass (patient image).

Proximal and distal control of the common, internal, and external carotid arteries was achieved. Carotid cross-clamping was then performed. A selective intraluminal shunt was not used, as cerebral oximetry values remained stable throughout clamping and satisfactory backflow was observed. Standard CEA was completed, and the arteriotomy was closed using a prosthetic patch angioplasty (Fig. 3). After clamp release, meticulous hemostasis was achieved. A single closed-suction drain was placed, and the cervical incision was closed in layers.

Activated clotting time was reassessed after the completion of the carotid procedure and measured approximately 250 seconds. As adequate anticoagulation was maintained, no additional heparin was administered. The surgical team then proceeded directly to the coronary phase.

Off-pump CABG was performed without cardiopulmonary bypass. The previously harvested LIMA was anastomosed to the LAD on the beating heart using standard stabilization techniques. The coronary revascularization was completed uneventfully, with no interval between the carotid and coronary procedures.

The patient was transferred to the intensive care unit in a stable condition. He was extubated 4 hours postoperatively and remained neurologically intact. Postoperative duplex ultrasonography confirmed a widely patent carotid reconstruction without residual stenosis.

## Discussion

The coexistence of severe CAS and critical coronary artery disease represents a complex clinical scenario in which perioperative cerebrovascular and myocardial ischemic risks must be carefully balanced. According to the 2023 ESVS guidelines, management of patients with concomitant carotid and coronary artery disease should be individualized, and synchronous CEA and CABG may be considered in selected high-risk patients^[^2^]^.

Several alternative management strategies may be considered in such patients, including staged CEA followed by CABG, staged carotid artery stenting prior to cardiac surgery, or isolated coronary revascularization with optimal medical therapy. However, staged approaches may expose patients to interval risks, including delayed myocardial revascularization or increased perioperative stroke risk, depending on the sequence selected. In appropriately selected patients and experienced centers, synchronous CEA and CABG has been shown to be a feasible strategy with acceptable perioperative outcomes^[^3^]^.

In the present case, a simultaneous approach was favored to address both vascular territories during a single anesthetic exposure. Carotid artery stenting or transcarotid artery revascularization were considered less suitable due to the presence of a retained metallic foreign body at the carotid bifurcation, which could increase embolic risk and technical complexity. Although minimally invasive direct coronary artery bypass (MID-CAB) may be feasible for isolated LAD disease, it was not favored in this case due to the need for a combined cervical and cardiac procedure, where median sternotomy provided superior exposure, operative control, and overall procedural safety. Isolated coronary revascularization alone was also deemed suboptimal given the presence of critical unilateral carotid stenosis.

An off-pump coronary artery bypass strategy was selected to minimize aortic manipulation and reduce embolic load in a patient with significant cerebrovascular vulnerability. Previous studies have demonstrated a reduction in early postoperative neurological complications, including stroke, with off-pump techniques in selected high-risk populations^[^4^]^. However, this strategy is not without limitations. Recent meta-analyses have reported higher rates of mid-term coronary reintervention and potential concerns regarding long-term mortality compared with on-pump CABG ^[^5^]^. These risks were carefully weighed during multidisciplinary decision-making, and the off-pump approach was selected based on an individualized risk–benefit assessment.

The presence of a long-standing retained ballistic fragment near the carotid bifurcation represents an exceptionally rare anatomical challenge. Chronic foreign bodies may induce dense fibrosis, obscure normal tissue planes, and increase the risk of cranial nerve injury during surgical dissection. The literature emphasizes the importance of meticulous preoperative imaging, secure vascular control, and careful handling of adjacent neurovascular structures in such cases^[^6^]^. In the present patient, adherence to these principles was essential to achieving a favorable outcome. This case emphasizes the importance of multidisciplinary decision-making and individualized surgical planning in complex patients with concomitant cerebrovascular and coronary disease.

### Patient perspective

The patient agreed to undergo synchronous surgery to minimize anesthetic exposure and expedite recovery. He reported an uneventful recovery and early return to activity without neurological symptoms.

### Learning points

- Management of patients with concomitant severe CAS and critical coronary artery disease should be individualized based on neurological status, anatomical complexity, and urgency of revascularization.
- Simultaneous CEA and off-pump CABG may be considered in carefully selected patients and experienced centers.
- Retained ballistic fragments near the carotid bifurcation significantly increase operative complexity and require meticulous preoperative imaging, careful neurovascular dissection, and secure vascular control.
